# Effectiveness of the Korean National Cancer Screening Program in Reducing Colorectal Cancer Mortality

**DOI:** 10.3390/cancers16244278

**Published:** 2024-12-23

**Authors:** Hyeon Ji Lee, Kyeongmin Lee, Byung Chang Kim, Jae Kwan Jun, Kui Son Choi, Mina Suh

**Affiliations:** 1 National Cancer Control Institute, National Cancer Center, Goyang 10408, Republic of Korea; leehj612@ncc.re.kr (H.J.L.);; 2 Department of Cancer Control and Policy, Graduate School of Cancer Science and Policy, National Cancer Center, Goyang 10408, Republic of Korea; 3 Center for Colorectal Cancer, Center for Cancer Prevention and Detection, National Cancer Center, Goyang 10408, Republic of Korea

**Keywords:** colorectal neoplasms, fecal immunochemical test, mass screening, early detection of cancer, cause of death

## Abstract

Background: Whether colorectal cancer (CRC) screening with a fecal immunochemical test (FIT) reduces mortality remains unclear. In South Korea, CRC screening with a FIT for individuals aged ≥ 50 years has been part of the Korean National Cancer Screening Program (KNCSP) since 2004. The aim of this study was to evaluate the effectiveness of the KNCSP in reducing CRC-specific mortality. Methods: We conducted a nested case-control study using cohort-based data derived from the KNCSP database. The cohort included 5,944,540 colorectal cancer-free individuals aged ≥ 50 years as of 2004. Individuals who died after CRC diagnosis were defined as cases (*n* = 29,992) and their sociodemographic characteristics were matched to those of the selected controls. The effects of screening exposure, frequency, and time interval on CRC-specific mortality were analyzed according to age group. Conditional logistic regression analysis was performed. Results: Compared with individuals who had never been screened, those who had ever been screened showed an OR of 0.74 (95% CI, 0.71–0.76) for CRC-specific mortality. CRC-specific mortality decreased as the number of screenings increased. Similar results were observed for those aged 50–79 years; however, the results for those aged 75–79 years were not statistically significant. Moreover, those aged ≥ 80 years had the opposite results. Conclusions: CRC mass screening using FIT is effective for individuals aged 50–74 years; therefore, this study suggests that countries considering introducing national CRC screening implement FIT for those within this age range.

## Simple Summary

There is limited evidence on the mortality reduction effect of colorectal cancer (CRC) screening using a fecal immunochemical test (FIT) from long-term follow-up nationwide cohort data. This nested case-control study demonstrated that the annual FIT conducted through the Korean National Cancer Screening Program reduced CRC-specific mortality in Koreans aged 50 to 74. Additionally, screening frequency and time interval also affected CRC-specific mortality reduction. Therefore, it is proposed that countries planning to implement national CRC screening programs consider the appropriate age for FIT.

## 1. Introduction

Colorectal cancer (CRC) is the third most commonly diagnosed cancer worldwide and the second leading cause of cancer-related death [1]. South Korea ranks third in cancer incidence and cancer-related mortality [2]. The prevalence of CRC is high in developed countries and tends to increase in transitional countries. By 2040, the expected number of new cases of CRC is 3.2 million, indicating a 63% increase from the 2 million cases reported in 2020. Moreover, the number of CRC-specific mortalities is expected to increase by 73.4% from 1 million in 2020 to 1.6 million by 2040 [3]. CRC stage at diagnosis is the most critical factor affecting survival outcome, and it takes 10–15 years for the precursor lesion to progress to CRC [4]. Screening is one of the most effective ways to reduce CRC-specific mortality by preventing the development of CRC and facilitating treatment in the early stages of cancer progression.

The incidence of CRC and the associated disease burden are expected to increase, highlighting the crucial role of early screening in managing CRC. Globally, interventions at a national level are being considered [3]. Colonoscopy and fecal occult blood tests (FOBTs) serve as screening modalities for the early detection of CRC. Colonoscopy helps detect abnormal lesions, remove precancerous lesions such as polyps, and prevent and treat colon cancer. Many studies have reported the impact of colonoscopy in reducing the incidence and mortality of CRC and increasing CRC survival rates. Colonoscopy is considered an effective and appropriate method for CRC screening [5,6,7,8]. FOBTs have a higher participation compliance rate than colonoscopy and can be used even with limited resources; therefore, they have high utility in mass screening [9,10,11]. Additionally, inspection accuracy has been further improved with the transition from traditional FOBTs to fecal immunochemical tests (FITs) [12,13,14,15,16]. FITs are also called immunochemical-FOBTs and are distinct from Guaiac-FOBTs (g-FOBTs), a traditional FOBT method. FITs are a method of detecting colorectal cancer using human hemoglobin, which has similar specificity to g-FOBTs in detecting advanced neoplasms, but with higher sensitivity and fewer false negatives than g-FOBTs [16,17,18]. The effectiveness of FOBT-based screening in detecting high-risk CRC has been demonstrated in previous studies. However, there is insufficient evidence regarding whether screening using the FOBT-based method reduces CRC mortality. In particular, studies confirming the long-term effects of early screening using FIT at the national level through population-based cohort studies are limited.

Since 2004, South Korea has implemented the CRC screening program as an integral component of the Korean National Cancer Screening Program (KNCSP), offering screening services to the entire population. From 2004 to 2012, FIT was provided as a primary screening method biannually to individuals aged 50 years or older. In 2012, the screening interval was changed to annually. If a FIT confirms an increased risk of CRC, additional colonoscopy tests are recommended [19]. Before 2023, a double-contrast barium enema was one of the secondary screening methods; it was excluded from the KNCSP due to decreased demand and changes in the medical environment. The KNCSP is conducted considering the Korean guidelines for colorectal cancer screening based on domestic and international medical and scientific evidence and the domestic medical environment, including finances [20,21,22,23].

The aim of this study was to evaluate the effect of CRC screening using FIT on mortality through cohort data of more than 10 years derived from the KNCSP database and to provide real-world evidence to countries considering the implementation of mass CRC screening.

## 2. Materials and Methods

### 2.1. Data Source

In this nested case-control study, we examined the effects of CRC screening on CRC-specific mortality using cohort-based data derived from the KNCSP, which has been conducted since 2004. The cohort was established by linking data from the KNCSP database, the Korea Central Cancer Registry (KCCR) database [2], and death certificate data from Statistics Korea based on the personal identification numbers of the individuals. The KNCSP data include a list of individuals invited for screening, their sociodemographic characteristics (sex, age, and economic level), and screening results. The KCCR database was used to confirm the history and incidence of CRC, and death certificate data were used to confirm the cause and date of death. Since the entire KNCSP data is managed by the National Health Insurance Service (NHIS), these data were linked and anonymized by the NHIS and provided to us for analysis as customized data.

To use the data, approval was required from the NHIS review committee based on the study proposal and ethics approval of the Institutional Review Board (IRB). The data used in these analyses were published and anonymized in accordance with confidentiality guidelines. Therefore, the requirement of informed consent from individuals was waived. This study was approved by the IRB of the National Cancer Center, Korea (IRB no. NCCNCS08129), and adhered to the principles of the Declaration of Helsinki.

### 2.2. Study Population

**Figure 1:**
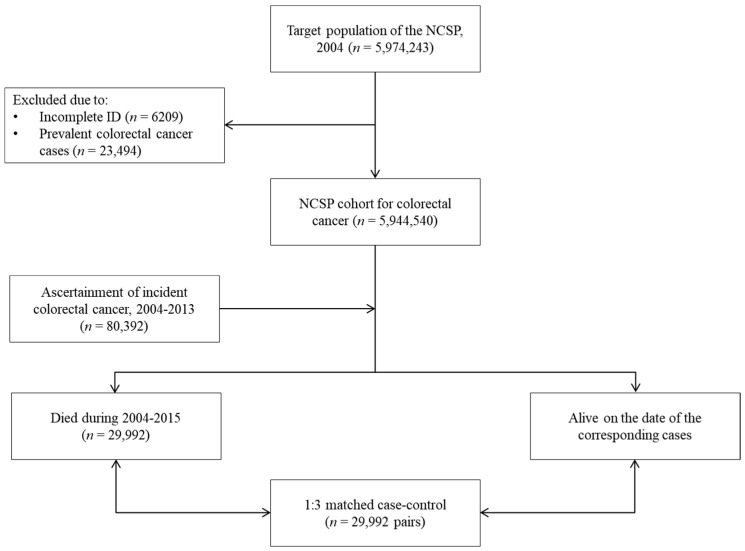
Flow chart for the selection of cases and controls; Among 5,994,549 identified individuals aged ≥ 50 years and without a history of colorectal cancer in 2004, 80,392 with newly diagnosed colorectal cancer during 2004–2013 were followed until 31 December 2015. In addition, 29,992 individuals who died from all causes during the follow-up period were selected as cases. Cases and controls were matched in a 1:3 ratio using year of entry, sex, age, and socioeconomic status as matching variables.

The baseline cohort of this study consisted of men and women aged ≥ 50 years (*n* = 5,974,243) who were invited to participate in the KNCSP in 2004 when CRC screening was included in the program. Among them, 6209 individuals with incomplete personal identification numbers and 23,494 with a history of CRC were excluded. Finally, in 2004, 5,944,540 cancer-free Korean men and women aged ≥ 50 years were included in the cohort (Figure 1). The cases and controls were classified based on death among the final cohort members. A case was defined as a person who died of all causes before the last day of December 2015 among those newly diagnosed with CRC (International Classification of Disease, 10th edition, codes C18-C20) [24] between 2004 and 2013. Based on incidence density sampling, three controls for each case were selected from all cohort individuals alive on the date of death in each case, using the cohort entry year, sex, age, and economic level as matching variables. The cohort entry year refers to the year the KNCSP-CRC screening was started; in other words, the year when one turned 50 years old. Using the NHIS beneficiaries’ income bracket [25], the economic level was divided into the following three groups: the upper 50% of beneficiaries were classified as the high-income group, the lower 50% as the middle-income group, and the medical aid recipients as the low-income group.

### 2.3. Measurements

The primary outcome variable in this study was CRC-specific mortality. The secondary outcome was all-cause mortality, except CRC-related mortality. The independent variable in this study was CRC screening exposure. For cases in matched pairs, the CRC diagnosis date was defined as the index date. In addition, starting from 1 January 2004, index dating was defined as the follow-up period for CRC screening, and a similar follow-up period was established between matched case-control pairs. All case-control pairs were invited to the KNCSP-CRC screening at least once and had the opportunity to undergo screening before the index date.

Additional analyses were performed according to the number of screening exposures and the time interval between CRC diagnosis and screening. During the screening follow-up period, those confirmed by the KNCSP in at least one screening were defined as the ever-screened group. In addition, the number of screening exposures during the follow-up period was categorized as once, twice, and three or more times. The time interval until diagnosis was defined as the period between the last screening date and index date and was divided into ≤11, 12–23, 24–35, 36–47, and ≥48 months.

### 2.4. Statistical Analysis

Conditional logistic regression analysis was conducted in this nested case-control study. The odds ratios (ORs) and 95% confidence intervals (CIs) for the outcome variables were calculated for individuals who had never undergone CRC screening on the index date. For all statistical analyses, statistical significance was set at *p* < 0.05. All statistical analyses were conducted using the SAS version 9.4 (SAS Institute, Cary, NC, USA) program.

## 3. Results

Between 2004 and 2013, 80,392 individuals were newly diagnosed with CRC. Among them, 29,992 died of all-cause mortality before the end of 2015 and 23,455 died of CRC. Table 1 presents the sociodemographic characteristics of the case-control pairs and the results of the association between CRC screening and mortality. Compared with individuals who had never been screened, those who had been screened exhibited an OR of 0.74 (95% CI 0.71–0.76) for CRC-specific mortality. The OR for all-cause mortality was 0.77, and 0.90 for all-cause mortality except CRC-specific mortality (Table 1). According to the analysis results stratified by age group, the OR for CRC-specific mortality was lower in younger age groups than in the older ones. However, the OR was higher in the ever-screened group than in the reference group for individuals aged ≥ 80 years. While the OR for CRC-specific mortality was lower than that of the reference group for those aged ≤ 79 years, it was not significant for those aged 75–79 years. The OR was higher than that of the reference group for those aged ≥ 80 years, and the results were statistically significant for the 80–84 years age group but not for those aged ≥ 85 years. The analysis results stratified by sex revealed no difference in CRC-specific mortality, with an OR of 0.73 for both men and women (95% CI, 0.70–0.77; 95% CI, 0.69–0.78, respectively) (Supplementary Table S1).

**Figure 2:**
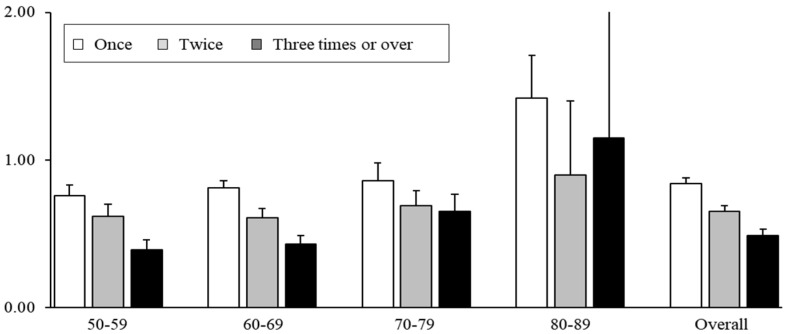
Association between frequency of screening and colorectal cancer mortality in ever-screened individuals compared with never-screened individuals. Conditional logistic regression results stratified by the number of screening exposures of each individual indicate that, as the number of screening exposures increased (once, twice, and three or more times), the risk of colorectal cancer-specific mortality decreased, except for individuals in their 80s.

**Figure 3:**
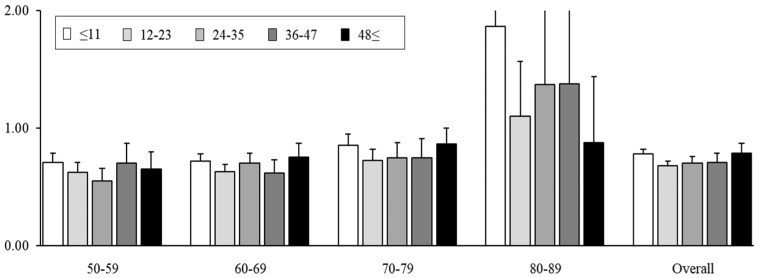
Association between time interval (months) since last screening and colorectal cancer mortality in ever-screened individuals compared with never-screened individuals; Conditional logistic regression results stratified by the time interval since the last screening of each individual showed that the time interval between colorectal cancer diagnosis and the last screening before this had a mortality reduction effect even beyond 48 months; it decreased further between 1 and 2 years. These results are significant only in individuals in their 50s and 70s, while this effect could not be confirmed in those aged over 80 years.

The results based on the number of screening exposures of each individual showed that as the number of screening exposures increased to once, twice, and three or more times, the OR for CRC-specific mortality significantly decreased to 0.84 (95% CI, 0.81–0.88), 0.65 (95% CI, 0.64–0.69), and 0.48 (95% CI, 0.44–0.53), respectively (Figure 2). The results based on the screening time interval from the index date to the last screening date showed ORs of 0.78 (95% CI, 0.74–0.82), 0.68 (95% CI, 0.63–0.72), 0.70 (95% CI, 0.65–0.76), 0.71 (95% CI, 0.64–0.79), and 0.79 (95% CI, 0.72–0.87) for ≤11, 12–23, 24–35, 36–47, and ≥48 months, respectively (Figure 3). Age group analysis showed similar trends in the 50s to 70s age groups; however, the 80s age group showed the opposite trend, with statistically insignificant results.

## 4. Discussion

This study was conducted to examine the effectiveness of a national CRC screening program using FITs on mortality reduction. The analysis showed a 26% reduction in CRC-specific mortality as a result of the screening. Previous studies have reported the effectiveness of screening using FITs in reducing CRC incidence and mortality and improving survival, emphasizing its role in reducing the CRC burden [24,26,27,28,29,30,31]. In particular, prior research has reported mortality reductions ranging from 10% to 40% [26,27,28,29]. Our results also demonstrated a decrease in mortality within the same range as that reported in previous studies. However, the effects of screening were observed only in certain age groups. CRC screening effectively reduced CRC mortality among individuals aged 50–74 years. However, the results were not statistically significant for those aged 75–79 years. Notably, the CRC mortality rate was high in the ever-screened group for individuals aged 80–84 years.

Furthermore, in the present study, the possibility of CRC-specific death decreased as the number of CRC screenings per individual increased, and the time interval between CRC diagnosis and the last screening contributed to a reduction in mortality, even beyond 48 months, decreasing further between 1 and 2 years. These results were significant only in individuals in their 50s and 70s, while these effects could not be confirmed in those over 80 years of age. This study holds significance as we analyzed the frequency of screening and test intervals through a population-based, long-term follow-up cohort. Although previous studies have highlighted the importance of repeated FITs [32,33], few studies have included population-based cohorts to evaluate the effects of the screenings on mortality. Achieving long-term effects requires high compliance with repeated tests [34], and this result highlights that FITs must be performed repeatedly within a relatively short and appropriate period.

Most CRC screening recommendations suggest appropriate screening targets, methods, and screening intervals through a systematic literature review, and modeling studies recommend annual or biannual screening using FIT for individuals aged 50–75 years [35]. In South Korea, although CRC screening involves a yearly FIT for individuals over 50 years of age, the National Cancer Screening Recommendation suggests performing FIT every 1 or 2 years for those aged 45–80 years [20]. Our findings add evidence to support CRC screening guidelines while demonstrating the need for discussion regarding the upper age limit for CRC screening eligibility. Based on the results of this study, performing CRC screening for individuals aged ≥ 80 years may be inefficient, and screening effectiveness remains inconclusive for those aged 75–79 years; therefore, consideration could be given to establishing an upper age limit for KNCSP-CRC screening.

We conducted a nationwide cohort study with a long-term follow-up to evaluate the effectiveness of CRC mass-screening programs. In addition, we used cancer registration and death certificate data with links based on systematic information from high-quality KNCSP invitations, attendance, and screening results. These findings are important because they provide real-world evidence for CRC mass screening.

Nevertheless, this study has some limitations. First, our study design was an observational rather than a randomized controlled trial. Therefore, the analysis results may be distorted owing to self-selection and lead-time biases. Although the KNCSP invites all eligible individuals for screening, those who respond to the invitation may represent a healthier subset or those aware of the importance of screening. Owing to this selection bias, the effectiveness of CRC screening may have been overestimated. Efforts were made to explain self-selection bias by performing an additional analysis of all-cause mortality, except CRC-specific mortality. However, no independent effects of CRC screening were observed. Second, several inherent methodological biases in the study design, such as misclassification, may have affected our results. Notably, a higher probability of death was observed within 11 months, except for those with an interval of over 48 months between the last CRC screening date and diagnosis. This may be attributed to the immediate diagnosis and subsequent death immediately after screening for the high-risk group because the KNCSP provides screening to the hidden average-risk group. Third, while opportunistic screening is available in South Korea [36], we only analyzed data of those who participated in the KNCSP. Therefore, the results of this analysis might have been underestimated. Finally, in addition to the matching variables, it was impossible to adjust for confounding variables that could have affected the results. CRC-related mortality can be influenced by lifestyle factors such as obesity and smoking [37,38,39,40]. Analysis was impossible because the corresponding information of individuals who did not participate in the KNCSP could not be identified.

The results of this study showed that the national CRC screening program using FITs in South Korea effectively reduced CRC mortality among individuals aged 50–74 years, and the probability of death decreased as the frequency of screening increased. However, a screening effect was not observed in individuals aged ≥ 75 years.

The FIT tends to be devalued as an ineffective CRC screening modality compared with colonoscopy. However, this study showed the effectiveness of the FIT in reducing mortality, particularly for specific age ranges, in national CRC screening for large populations. The FIT may be a more practical modality for mass screening, as it is relatively inexpensive and less susceptible to physician proficiency, and could thus ensure standardized screening results.

## 5. Conclusions

In conclusion, this study proposes introducing CRC screening using FITs in countries that hesitate to introduce national CRC screening. Notably, the age range of the screening target population should also be considered to enhance screening efficiency.

## Figures and Tables

**Table 1:** Association between receipt of colorectal cancer screening and cause of mortality.

	All-Cause Mortality					Colorectal Cancer-Specific Mortality					All-Cause Mortality Except from Colorectal Cancer				
		Screened, %					Screened, %					Screened, %			
	Pairs, *n*	Case	Control	OR	95% CI	Pairs, *n*	Case	Control	OR	95% CI	Pairs, *n*	Case	Control	OR	95% CI
Overall	29,992	23.50	27.70	0.77	0.74–0.79	23,455	23.60	28.60	0.74	0.71–0.76	6537	23.10	24.80	0.90	0.83–0.96
Sex															
Male	18,587	26.00	30.50	0.77	0.74–0.80	14,122	26.00	31.40	0.73	0.70–0.77	4465	26.00	27.60	0.91	0.83–0.99
Female	11,405	19.40	23.30	0.75	0.71–0.85	9333	19.90	24.30	0.73	0.69–0.78	2072	17.00	18.80	0.86	0.74–1.00
10-year age groups															
50–59	6072	27.80	24.80	0.68	0.64–0.73	5201	27.30	34.90	0.66	0.61–0.71	871	30.70	34.10	0.83	0.70–1.00
60–69	10,411	29.90	36.10	0.71	0.68–0.75	8013	30.20	37.30	0.69	0.65–0.73	2398	28.80	32.40	0.82	0.73–0.91
70–79	10,153	19.30	21.70	0.84	0.79–0.89	7631	19.20	22.50	0.79	0.74–0.85	2522	19.40	19.40	0.99	0.88–1.12
80+	3356	8.40	6.70	1.29	1.10–1.50	2610	8.40	6.60	1.31	1.10–1.55	746	8.60	7.10	1.22	0.88–1.68
5-year age groups															
50–54	3100	25.90	32.40	0.69	0.63–0.76	2723	26.20	32.50	0.70	0.63–0.78	377	23.90	31.80	0.63	0.47–0.84
55–59	2972	29.80	37.20	0.67	0.61–0.74	2478	28.60	37.50	0.62	0.55–0.69	494	35.80	35.90	1.00	0.80–1.25
60–64	5348	30.70	38.40	0.66	0.62–0.71	4186	30.70	39.20	0.64	0.59–0.69	1162	30.60	35.50	0.77	0.66–0.90
65–69	5063	29.10	33.70	0.77	0.71–0.83	3827	29.70	35.10	0.74	0.68–0.81	1236	27.10	29.50	0.87	0.74–1.02
70–74	6491	21.60	25.00	0.80	0.74–0.86	4852	21.60	26.10	0.75	0.69–0.82	1639	21.50	21.70	0.98	0.85–1.14
75–79	3662	15.20	15.90	0.93	0.84–1.04	2779	15.10	16.20	0.91	0.80–1.03	883	15.60	15.20	1.02	0.82–1.27
80–84	2624	9.80	7.50	1.35	1.15–1.59	2048	9.60	7.40	1.38	1.15–1.65	576	10.20	8.00	1.27	0.90–1.79
85+	732	3.70	3.00	1.20	0.69–2.08	562	3.90	3.00	1.31	0.71–2.41	170	2.90	3.00	0.79	0.20–3.11
Economic level															
High	16,187	20.60	25.60	0.70	0.67–0.74	12,556	20.90	26.30	0.69	0.65–0.72	3631	19.50	22.90	0.77	0.70–0.86
Middle	9227	27.50	32.10	0.77	0.73–0.82	7375	27.40	33.00	0.73	0.68–0.77	1852	28.00	28.50	0.98	0.86–1.11
Low	4578	25.80	26.60	0.96	0.88–1.04	3524	25.40	27.10	0.91	0.82–1.00	1054	27.00	24.50	1.15	0.97–1.37

Analyses were performed on 1-to-3 matched case-control sets using conditional logistic regression analysis and compared with never-screened individuals; CI, confidence interval; OR, odds ratio.

## Data Availability

This study used data from the customized health claim database provided by the National Health Insurance Service (NHIS). The NHIS review committee approves data use based on the study proposal and ethical approval of the Institutional Review Board. Data requests can be accessed from the National Health Insurance Data-Sharing Service home page (https://nhiss.nhis.or.kr accessed on 23 December 2024). Further inquiries about the use of data can be obtained by contacting the corresponding author.

## Supplementary Materials

The following supporting information can be downloaded at: https://www.mdpi.com/article/10.3390/cancers16244278/s1, Table S1: Association between receipt of colorectal cancer screening and cause of mortality stratified by sex.

## References

[1] Sung H., Ferlay J., Siegel R.L., Laversanne M., Soerjomataram I., Jemal A., Bray F. (2021). Global cancer statistics 2020: GLOBOCAN estimates of incidence and mortality worldwide for 36 cancers in 185 countries. CA Cancer J. Clin..

[2] Kang M.J., Jung K.-W., Bang S.H., Choi S.H., Park E.H., Yun E.H., Kim H.-J., Kong H.-J., Im J.-S., Seo H.G. (2023). Cancer statistics in Korea: Incidence, mortality, survival, and prevalence in 2020. Cancer Res. Treat..

[3] Morgan E., Arnold M., Gini A., Lorenzoni V., Cabasag C., Laversanne M., Vignat J., Ferlay J., Murphy N., Bray F. (2023). Global burden of colorectal cancer in 2020 and 2040: Incidence and mortality estimates from GLOBOCAN. Gut.

[4] Winawer S.J. (1999). Natural history of colorectal cancer. Am. J. Med..

[5] Zheng S., Schrijvers J.J., Greuter M.J., Kats-Ugurlu G., Lu W., de Bock G.H. (2023). Effectiveness of Colorectal Cancer (CRC) Screening on All-Cause and CRC-Specific Mortality Reduction: A Systematic Review and Meta-Analysis. Cancers.

[6] Levin T.R., Corley D.A., Jensen C.D., Schottinger J.E., Quinn V.P., Zauber A.G., Lee J.K., Zhao W.K., Udaltsova N., Ghai N.R. (2018). Effects of organized colorectal cancer screening on cancer incidence and mortality in a large community-based population. Gastroenterology.

[7] Lauby-Secretan B., Vilahur N., Bianchini F., Guha N., Straif K. (2018). The IARC perspective on colorectal cancer screening. N. Engl. J. Med..

[8] Guo F., Chen C., Holleczek B., Schöttker B., Hoffmeister M., Brenner H. (2021). Strong reduction of colorectal cancer incidence and mortality after screening colonoscopy: Prospective cohort study from Germany. Am. J. Gastroenterol..

[9] Almog R., Ezra G., Lavi I., Rennert G., Hagoel L. (2008). The public prefers fecal occult blood test over colonoscopy for colorectal cancer screening. Eur. J. Cancer Prev..

[10] Sobhani I., Alzahouri K., Ghout I., Charles D.J., Durand-Zaleski I. (2011). Cost-effectiveness of mass screening for colorectal cancer: Choice of fecal occult blood test and screening strategy. Dis. Colon Rectum.

[11] Lopes G., Stern M.C., Temin S., Sharara A.I., Cervantes A., Costas-Chavarri A., Engineer R., Hamashima C., Ho G.F., Huitzil F.D. (2019). Early detection for colorectal cancer: ASCO resource-stratified guideline. J. Glob. Oncol..

[12] Clark G., Strachan J.A., Carey F.A., Godfrey T., Irvine A., McPherson A., Brand J., Anderson A.S., Fraser C.G., Steele R.J. (2021). Transition to quantitative faecal immunochemical testing from guaiac faecal occult blood testing in a fully rolled-out population-based national bowel screening programme. Gut.

[13] Shapiro J.A., Bobo J.K., Church T.R., Rex D.K., Chovnick G., Thompson T.D., Zauber A.G., Lieberman D., Levin T.R., Joseph D.A. (2017). A comparison of fecal immunochemical and high-sensitivity guaiac tests for colorectal cancer screening. Am. J. Gastroenterol..

[14] Tinmouth J., Lansdorp-Vogelaar I., Allison J.E. (2015). Faecal immunochemical tests versus guaiac faecal occult blood tests: What clinicians and colorectal cancer screening programme organisers need to know. Gut.

[15] Levi Z., Birkenfeld S., Vilkin A., Bar-Chana M., Lifshitz I., Chared M., Maoz E., Niv Y. (2011). A higher detection rate for colorectal cancer and advanced adenomatous polyp for screening with immunochemical fecal occult blood test than guaiac fecal occult blood test, despite lower compliance rate. A prospective, controlled, feasibility study. Int. J. Cancer.

[16] Mousavinezhad M., Majdzadeh R., Sari A.A., Delavari A., Mohtasham F. (2016). The effectiveness of FOBT vs. FIT: A meta-analysis on colorectal cancer screening test. Med. J. Islam. Repub. Iran.

[17] Allison J.E., Tekawa I.S., Ransom L.J., Adrain A.L. (1996). A comparison of fecal occult-blood tests for colorectal-cancer screening. N. Engl. J. Med..

[18] Meklin J., Syrjänen K., Eskelinen M. (2020). Fecal occult blood tests in colorectal cancer screening: Systematic review and meta-analysis of traditional and new-generation fecal immunochemical tests. Anticancer Res..

[19] Park B., Her E.Y., Lee K., Nari F., Jun J.K., Choi K.S., Suh M. (2023). Overview of the National Cancer Screening Program for Colorectal Cancer in Korea over 14 Years (2004–2017). Cancer Res. Treat..

[20] Sohn D.K., Kim M.J., Park Y., Suh M., Shin A., Lee H.Y., Im J.P., Cho H.-M., Hong S.P., Kim B.-h. (2015). The Korean guideline for colorectal cancer screening. JKMA J. Korean Med. Assoc..

[21] Shin A., Choi K.S., Jun J.K., Noh D.K., Suh M., Jung K.-W., Kim B.C., Oh J.H., Park E.-C. (2013). Validity of fecal occult blood test in the national cancer screening program, Korea. PLoS ONE.

[22] Park B., Lee Y.Y., Song S.Y., Shin H.Y., Suh M., Choi K.S., Jun J.K. (2022). Trends of colorectal cancer screening rates in Korea: Korean national cancer screening survey 2005–2020. Gut Liver.

[23] Lee B.H., Jeong S.Y. (2002). Korean national recommendation guidelines on screening and surveillance for early detection of colorectal cancers. J. Korean Med. Assoc..

[24] Luu X.Q., Lee K., Jun J.K., Suh M., Jung K.W., Choi K.S. (2022). Effect of colorectal cancer screening on long-term survival of colorectal cancer patients: Results of the Korea National Cancer Screening Program. Int. J. Cancer.

[25] Ahn E. (2020). Introducing big data analysis using data from National Health Insurance Service. Korean J. Anesthesiol..

[26] Zappa M., Castiglione G., Grazzini G., Falini P., Giorgi D., Paci E., Ciatto S. (1997). Effect of faecal occult blood testing on colorectal mortality: Results of a population-based case-control study in the district of Florence, Italy. Int. J. Cancer.

[27] Gini A., Jansen E.E., Zielonke N., Meester R.G., Senore C., Anttila A., Segnan N., Mlakar D.N., de Koning H.J., Lansdorp-Vogelaar I. (2020). Impact of colorectal cancer screening on cancer-specific mortality in Europe: A systematic review. Eur. J. Cancer.

[28] Rossi P.G., Vicentini M., Sacchettini C., Di Felice E., Caroli S., Ferrari F., Mangone L., Pezzarossi A., Roncaglia F., Campari C. (2015). Impact of screening program on incidence of colorectal cancer: A cohort study in Italy. Am. J. Gastroenterol..

[29] Chiu H.M., Chen S.L.S., Yen A.M.F., Chiu S.Y.H., Fann J.C.Y., Lee Y.C., Pan S.L., Wu M.S., Liao C.S., Chen H.H. (2015). Effectiveness of fecal immunochemical testing in reducing colorectal cancer mortality from the One Million Taiwanese Screening Program. Cancer.

[30] Hewitson P., Glasziou P., Watson E., Towler B., Irwig L. (2008). Cochrane systematic review of colorectal cancer screening using the fecal occult blood test (hemoccult): An update. Am. J. Gastroenterol..

[31] Zorzi M., Fedeli U., Schievano E., Bovo E., Guzzinati S., Baracco S., Fedato C., Saugo M., Dei Tos A.P. (2015). Impact on colorectal cancer mortality of screening programmes based on the faecal immunochemical test. Gut.

[32] Murphy C.C., Sen A., Watson B., Gupta S., Mayo H., Singal A.G. (2020). A systematic review of repeat fecal occult blood tests for colorectal cancer screening. Cancer Epidemiol. Biomark..

[33] Holme Ø., Bretthauer M., Fretheim A., Odgaard-Jensen J., Hoff G. (2013). Flexible sigmoidoscopy versus faecal occult blood testing for colorectal cancer screening in asymptomatic individuals. Cochrane Database Syst. Rev..

[34] Hassan C., Rossi P.G., Camilloni L., Rex D., Jimenez-Cendales B., Ferroni E., Borgia P., Zullo A., Guasticchi G., Group H. (2012). Meta-analysis: Adherence to colorectal cancer screening and the detection rate for advanced neoplasia, according to the type of screening test. Aliment. Pharmacol. Ther..

[35] Bénard F., Barkun A.N., Martel M., von Renteln D. (2018). Systematic review of colorectal cancer screening guidelines for average-risk adults: Summarizing the current global recommendations. World J. Gastroenterol..

[36] Hong S., Lee Y.Y., Lee J., Kim Y., Choi K.S., Jun J.K., Suh M. (2021). Trends in cancer screening rates among Korean men and women: Results of the Korean National Cancer Screening Survey, 2004–2018. Cancer Res. Treat..

[37] Shaukat A., Dostal A., Menk J., Church T.R. (2017). BMI is a risk factor for colorectal cancer mortality. Dig. Dis. Sci..

[38] Murphy T.K., Calle E.E., Rodriguez C., Kahn H.S., Thun M.J. (2000). Body mass index and colon cancer mortality in a large prospective study. Am. J. Epidemiol..

[39] Chao A., Thun M.J., Jacobs E.J., Henley S.J., Rodriguez C., Calle E.E. (2000). Cigarette smoking and colorectal cancer mortality in the cancer prevention study II. J. Natl. Cancer Inst..

[40] Botteri E., Iodice S., Bagnardi V., Raimondi S., Lowenfels A.B., Maisonneuve P. (2008). Smoking and colorectal cancer: A meta-analysis. JAMA.

