# Supplementary material for: Effectiveness of the Korean National Cancer Screening Program in Reducing Colorectal Cancer Mortality
Source: Cancers (Basel). 2024 Dec 23;16(24):4278. doi: 10.3390/cancers16244278 (PMC11674503; doi:10.3390/cancers16244278)
Supplement: Supplementary file 1 [file cancers-16-04278-s001.zip › cancers-3371182-supplementary.pdf]

Supplementary Table S1. Association between receipt of colorectal cancer screening and cause of mortality stratified by sex

	All-cause mortality					Colorectal cancer-specific mortality					All-cause mortality except from colorectal cancer				
		Screened, %					Screened, %					Screened, %			
	Pairs, n	Case	Control	OR	95% CI	Pairs, n	Case	Control	OR	95% CI	Pairs, n	Case	Control	OR	95% CI
Male	18,587	26.01	30.46	0.77	0.74-0.80	14,122	26.02	31.35	0.73	0.70-0.77	4465	25.98	27.61	0.91	0.83-0.99
10-year age groups															
50-59	4506	27.45	33.41	0.72	0.66-0.78	3787	26.78	33.43	0.69	0.63-0.75	719	31.02	33.32	0.89	0.73-1.08
60-69	7200	30.65	37.01	0.71	0.67-0.76	5372	30.73	38.19	0.67	0.62-0.72	1828	30.42	33.55	0.84	0.74-0.95
70-79	5468	22.40	24.63	0.86	0.80-0.93	3916	22.52	25.70	0.81	0.74-0.89	1552	22.10	21.93	1.00	0.86-1.17
80+	1413	11.68	9.43	1.27	1.04-1.56	1047	12.13	9.32	1.36	1.08-1.71	366	10.38	9.75	1.02	0.67-1.57
5-year age groups															
50-54	2267	24.79	30.42	0.72	0.65-0.81	1963	25.01	30.57	0.73	0.64-0.82	304	23.36	29.42	0.70	0.51-0.97
55-59	2239	30.15	36.45	0.71	0.64-0.80	1824	28.67	36.51	0.65	0.57-0.74	415	36.63	36.17	1.02	0.80-1.31
60-64	3817	31.12	38.95	0.66	0.61-0.72	2907	30.89	39.83	0.63	0.57-0.69	910	31.87	36.14	0.80	0.67-0.95
65-69	3383	30.12	34.83	0.77	0.70-0.84	2465	30.55	36.27	0.73	0.65-0.81	918	28.98	30.97	0.89	0.75-1.07
70-74	3744	24.28	26.92	0.85	0.77-0.93	2678	24.50	28.28	0.79	0.71-0.88	1066	23.73	23.49	1.01	0.85-1.21
75-79	1724	18.33	19.64	0.90	0.78-1.04	1238	18.26	20.10	0.87	0.73-1.03	486	18.52	18.47	0.98	0.75-1.29
80-84	1159	12.94	10.18	1.32	1.06-1.63	856	13.32	10.16	1.40	1.09-1.79	303	11.88	10.25	1.09	0.70-1.69
85+	254	5.91	4.74	1.35	0.61-3.02	191	6.81	4.31	1.82	0.74-4.45	63	3.17	6.20	0.38	0.04-3.41
Economic level															
High	10,776	22.97	28.18	0.71	0.68-0.76	8126	23.23	29.11	0.69	0.64-0.73	2650	22.15	25.29	0.81	0.72-0.91
Middle	5814	30.29	35.10	0.77	0.72-0.83	4542	29.63	35.77	0.72	0.67-0.78	1272	32.63	32.71	1.00	0.86-1.15
Low	1997	29.94	29.28	1.04	0.92-1.18	1454	30.26	30.12	1.01	0.88-1.16	543	29.10	26.98	1.15	0.91-1.45
Female	11,405	19.38	23.30	0.75	0.71-0.80	9333	19.91	24.30	0.73	0.69-0.78	2072	16.99	18.77	0.86	0.74-1.00
10-year age groups															
50-59	1566	28.86	38.70	0.59	0.51-0.67	1414	28.85	38.80	0.59	0.51-0.68	152	28.95	37.72	0.59	0.38-0.94
60-69	3211	28.25	34.14	0.72	0.65-0.79	2641	29.23	35.33	0.72	0.65-0.80	570	23.68	28.65	0.73	0.57-0.93
70-79	4685	15.65	18.34	0.81	0.73-0.89	3715	15.77	19.09	0.77	0.69-0.86	970	15.15	15.46	0.97	0.78-1.21
80+	1943	6.07	4.75	1.31	1.04-1.64	1563	5.89	4.79	1.25	0.96-1.61	380	6.84	4.61	1.56	0.94-2.59
5-year age groups															
50-54	833	28.93	37.94	0.62	0.51-0.74	760	29.21	37.59	0.64	0.53-0.78	73	26.03	41.55	0.40	0.20-0.79
55-59	733	28.79	39.56	0.56	0.46-0.68	654	28.44	40.21	0.53	0.43-0.65	79	31.65	34.18	0.86	0.46-1.61
60-64	1531	29.65	36.99	0.67	0.58-0.77	1279	30.34	37.79	0.67	0.57-0.78	252	26.19	32.94	0.66	0.47-0.95
65-69	1680	26.96	31.55	0.77	0.68-0.88	1362	28.19	33.02	0.77	0.67-0.89	318	21.70	25.26	0.79	0.57-1.10
70-74	2747	17.87	22.34	0.73	0.65-0.82	2174	18.03	23.37	0.70	0.61-0.79	573	17.28	18.39	0.92	0.70-1.19
75-79	1938	12.49	12.67	0.98	0.83-1.15	1541	12.59	13.04	0.96	0.80-1.15	397	12.09	11.22	1.10	0.76-1.59
80-84	1465	7.24	5.44	1.40	1.09-1.79	1192	6.96	5.39	1.35	1.03-1.78	273	8.42	5.63	1.62	0.94-2.79
85+	478	2.51	2.21	1.07	0.49-2.32	371	2.43	2.43	0.98	0.42-2.32	107	2.80	1.44	1.59	0.25-9.95
Economic level															
High	5411	15.75	20.44	0.67	0.61-0.74	4430	16.50	21.28	0.68	0.61-0.75	981	12.33	16.64	0.64	0.51-0.81
Middle	3413	22.77	26.97	0.76	0.68-0.84	2833	23.76	28.55	0.74	0.66-0.82	580	17.93	19.16	0.92	0.69-1.22
Low	2581	22.51	24.53	0.88	0.79-0.99	2070	21.93	25.03	0.83	0.73-0.94	511	24.85	22.53	1.15	0.88-1.50

Analyses were performed on 1-to-3 matched case-control sets using conditional logistic regression analysis and compared with never-screened individuals; CI, confidence interval; OR, odds ratio
